# Predicting response to physiotherapy treatment for musculoskeletal shoulder pain: a systematic review

**DOI:** 10.1186/1471-2474-14-203

**Published:** 2013-07-08

**Authors:** Rachel Chester, Lee Shepstone, Helena Daniell, David Sweeting, Jeremy Lewis, Christina Jerosch-Herold

**Affiliations:** 1 School of Allied Health Professions, Faculty of Medicine and Health Sciences, University of East Anglia, Norwich, Norfolk NR4 7TJ, UK; 2 Physiotherapy Department, Norfolk and Norwich University Hospital, Norwich, Norfolk NR4 7TJ, UK; 3 Norwich Medical School, Faculty of Medicine and Health Sciences, University of East Anglia, Norwich, Norfolk NR4 7TJ, UK; 4 Physiotherapy Department, East Coast Community Healthcare CIC, Lowestoft Hospital, Tennyson Road, Lowestoft, Suffolk NR32 1PT, UK; 5 School of Health and Social Work, Department of Allied Health Professions, University of Hertfordshire, College Lane, Hatfield AL10 9AB, UK; 6 Musculoskeletal Department, Health at the Stowe, Central London Community Healthcare, 260 Harrow Rd, Greater London, W2 5ES, UK

**Keywords:** Physical therapy, Shoulder, Shoulder pain, Musculoskeletal, Predict, Prognosis

## Abstract

**Background:**

People suffering from musculoskeletal shoulder pain are frequently referred to physiotherapy. Physiotherapy generally involves a multimodal approach to management that may include; exercise, manual therapy and techniques to reduce pain. At present it is not possible to predict which patients will respond positively to physiotherapy treatment. The purpose of this systematic review was to identify which prognostic factors are associated with the outcome of physiotherapy in the management of musculoskeletal shoulder pain.

**Methods:**

A comprehensive search was undertaken of Ovid Medline, EMBASE, CINAHL and AMED (from inception to January 2013). Prospective studies of participants with shoulder pain receiving physiotherapy which investigated the association between baseline prognostic factors and change in pain and function over time were included. Study selection, data extraction and appraisal of study quality were undertaken by two independent assessors. Quality criteria were selected from previously published guidelines to form a checklist of 24 items. The study protocol was prospectively registered onto the International Prospective Register of Systematic Reviews.

**Results:**

A total of 5023 titles were retrieved and screened for eligibility, 154 articles were assessed as full text and 16 met the inclusion criteria: 11 cohort studies, 3 randomised controlled trials and 2 controlled trials. Results were presented for the 9 studies meeting 13 or more of the 24 quality criteria. Clinical and statistical heterogeneity resulted in qualitative synthesis rather than meta-analysis. Three studies demonstrated that high functional disability at baseline was associated with poor functional outcome (p ≤ 0.05). Four studies demonstrated a significant association (p ≤ 0.05) between longer duration of shoulder pain and poorer outcome. Three studies, demonstrated a significant association (p ≤ 0.05) between increasing age and poorer function; three studies demonstrated no association (p > 0.05).

**Conclusion:**

Associations between prognostic factors and outcome were often inconsistent between studies. This may be due to clinical heterogeneity or type II errors. Only two baseline prognostic factors demonstrated a consistent association with outcome in two or more studies; duration of shoulder pain and baseline function. Prior to developing a predictive model for the outcome of physiotherapy treatment for shoulder pain, a large adequately powered prospective cohort study is required in which a broad range of prognostic factors are incorporated.

## Background

Shoulder pain has a lifetime prevalence of one in three [1] and is the third most common musculoskeletal condition presenting in primary care [2]. However just 50% of people referred to primary care with first episode shoulder pain show complete recovery within six months, rising to only sixty percent after one year [3].

Shoulder pain is one of the most common musculoskeletal disorders in the working population [4]. In 2011–2012, for the first time in Great Britain, the prevalence of work related upper limb disorders exceeded those of low back pain [5].

The most effective treatment for musculoskeletal shoulder pain is not known. Reports indicate that up to one third of patients referred to physiotherapy musculsoskeletal outpatient services have shoulder pain [6]. However clear indicators of who will and will not respond favourably to physiotherapy treatment is currently unavailable. When physiotherapy is unsuccessful, other interventions are often considered. However for some patients, the time spent in an unsuccessful course of physiotherapy may delay referral along another, possibly more appropriate pathway. This increases the likelihood of chronic pain and reduces the effectiveness of future interventions [5].

The exact cost of shoulder pain to healthcare and the economy is unclear. Studies in the Netherlands [7] and Sweden [8] have demonstrated that 12 [7] to 22 [8] percent of patients who visit primary care with shoulder pain incur between 74 and 91 percent of the total cost respectively; a relatively small percentage of patients incur a high percentage of the cost. This suggests that for some patients there may be a more effective and efficient management pathway for the resolution of shoulder pain. Between 47 [7] and 84 percent [8] of the total incurred cost is related to sickness absence. These same studies demonstrated that physiotherapy accounted for between 37 percent [7] and 60 percent [8] of the mean total healthcare cost. Those patients that used direct access to physiotherapy had lower healthcare and overall costs to the economy [8]. This comparatively low cost, non-invasive resource is therefore an obvious choice as a first line treatment for shoulder pain. However, a greater knowledge of prognostic factors in terms of who is likely to respond to physiotherapy and who will not is vital for patients, healthcare professionals and commissioners and ensures effective and efficient use of limited resources. Referral to physiotherapy for patients who respond favourably will be of considerable benefit. However for those patients who do not respond favourably to physiotherapy, delayed referral along a more effective pathway may be costly. A review of previous research has suggested that a range of biopsychosocial factors are related to outcome following General Practitioner management of shoulder pain [9]. The objective of this systematic review was to identify which prognostic factors are associated with the outcome from physiotherapy treatment for musculoskeletal shoulder pain. Primary outcomes of interest were functional recovery and pain over any time period.

## Methods

A systematic review was undertaken. The study protocol was published in advance and may be viewed on the International prospective register of systematic reviews (PROSPERO) (Submitted 21 December 2011, Registration number CRD42011001719, http://www.crd.york.ac.uk/PROSPERO/display_record.asp?ID=CRD42011001719).

PRISMA (Preferred Reporting Items for Systematic Reviews and Meta-analysis) [10] guidelines were followed.

### Search strategy

Medline, EMBASE, CINAHL and AMED were searched via Ovid using the NHS electronic library from inception to January 2013 using medical subject headings (MeSH), text terms and Boolean operators (RC). The full Medline search strategy is presented in Additional file 1. Search terms were adapted for the other databases. No language limits were applied. Reference lists of eligible publications were hand searched.

### Study selection

Two independent reviewers (RC and DS, RC and HD) evaluated all retrieved titles, and abstracts if required, against the pre-defined eligibility criteria. All potentially eligible publications were retrieved in full text and independently evaluated by two reviewers (RC and DS; RC and HD).

To be included in this review study participants had to have received physiotherapy for the management of musculoskeletal shoulder pain. Reports had to be published, at least in part, in a peer reviewed journal.

#### Study design

Prospective studies, of the following designs were included: i) Longitudinal cohort studies ii) controlled trials which carried out a subgroup analysis relating outcome in one or more arm of physiotherapy treatment to baseline variables and iii) controlled trials in which two or more groups of subjects, different at baseline, received the same physiotherapy treatment/package.

Controlled trials in which two or more groups of participants received (i) different forms of management, not all of which were physiotherapy, and (ii) prognostic factors were presented for all participants, such that prognostic factors for physiotherapy were not differentiated from those for other treatment group(s), were not included. Studies in which retrospective collection of prognostic factors took place were not included.

#### Participants

Studies could include participants of any age, with musculoskeletal shoulder pain of any duration. Studies in which more than 20% of participants presented post operatively, post fracture or traumatic dislocation or with pathologies or syndromes which referred directly to the shoulder from other regions were excluded. Studies that included anatomical regions in addition to the shoulder but did not report results for the shoulder as a distinct anatomical region were excluded.

#### Physiotherapy interventions

Participants must have received at least one session of physiotherapy, delivered by a physiotherapist and involving some direct clinical contact. Ideally all participants should have received a full course of physiotherapy; however this was likely to exclude a high proportion of valuable studies.

#### Prognostic factors

Potential prognostic factors had to be collected at baseline and had to include one or more of the following; individual participant characteristics, lifestyle, psychosocial factors, past experience and expectations of physiotherapy, shoulder symptoms and general health, signs of impairment from the objective/clinical examination, activity and participation, radiological imaging. Blood tests, surgical and arthroscopic findings are not usually undertaken prior to commencing physiotherapy and were therefore not considered as prognostic factors in this review.

#### Outcome measures

Studies which included any of the following outcome measures at any time point (including time to resolution of outcome) were included; pain, functional/disability scores measured by self-administered validated questionnaires, adverse events, Constant score [11], quality of life scores, return to work/days off work, range of shoulder movement and shoulder strength.

### Data extraction

Data from each included study were entered onto a custom designed data extraction form (Additional file 2) by two independent reviewers (RC and DS; RC and HD). The form was developed by RC, pilot tested by RC and DS on five studies, and after discussion with all reviewers, refined accordingly. The form included criteria relating to study design and setting, participant characteristics, physiotherapy treatment details, outcome measures and prognostic factors as well as factors relating to study quality and risk of bias. When more than one published paper reported results for the same group of participants all were utilized to gain information. If further clarity was required, attempts were made to contact the original authors.

### Quality assessment of external validity, risk of bias, and presentation of results

To the authors’ knowledge there is as yet no recommended validated tool for the assessment of quality in reviews of prognostic research using a variety of study designs. In addition, none of the tools identified assessed all the criteria necessary to address the objective of our review. Selection of criteria were therefore based on guidelines published by Hayden et al. [12], Downs and Black [13], the Newcastle Ottowa Score [14], relevant PEDro items [15], criteria previously used by Kuijpers [9] and additional clinical items which may have presented a risk of bias or limit the transferability of findings. These criteria formed a checklist (Additional file 3), each item being referenced to their original source(s), and against which each study was independently assessed by two reviewers (RC and DS; RC and HD). Twenty four items covering 3 domains were included; transferability of findings (A, B, C, L-P), potential for bias (D-P) and reporting quality (Q-Y).

## Results

### Study selection

**Figure 1:**
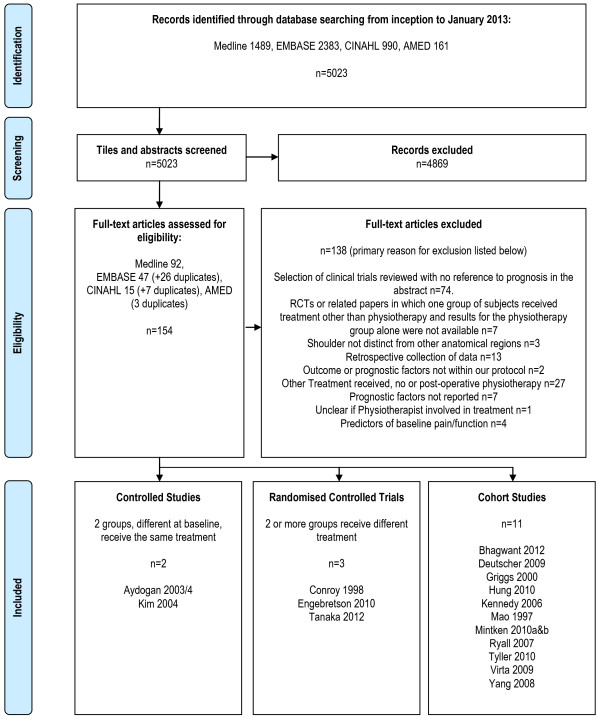
PRISMA Flow chart outlining the literature search and study selection.

The results of the search strategy are presented in the PRISMA flow diagram in Figure 1. A total of 16 publications were included in the final review. One study included more than one anatomical region and assessed prognostic indicators for conservative management generally rather than physiotherapy specifically [16]. One of the authors revisited study data specifically for this review and provided results for those participants with shoulder pain who had received physiotherapy [Personal communications: Palmer K and Ntani G, University of Southampton, 2012].

### Summary measures

Results are presented for each study and grouped according to outcome measure. Where results are presented in different formats within the same subheadings or full details omitted, this is because further details were unavailable.

Where available all statistical details of multiple regression analysis are tabulated. In view of the high number of potential prognostic factors investigated on univariate analysis and the variation in the measurement tools and categories used, full statistical details of univariate analysis are not included. Instead, to aid comparison between studies, prognostic factors which were investigated but not statistically significant within the final multiple regression analysis are listed and divided into two sections based on whether or not the probability of a random error on univariate analysis was 10% or less.

For studies that divided participants into two or more groups according to i) baseline characteristics [17,18] or ii) successful versus unsuccessful outcome [19], mean differences plus standard deviation and/or 95% confidence intervals (CI) for each group, and if available between groups are presented. Where studies have performed accuracy statistics for a clinical prediction rule, details of the former are presented [20,21].

In view of heterogeneity on a number of levels (study design, characteristics of shoulder pain, physiotherapy treatment, prognostic factors, outcome factors and selection of measurement tools), this review provides a best evidence synthesis rather than meta-analysis. Predictive factors demonstrated to have a statistically significant association with outcome on multiple regression analysis (or equivalent) in two or more studies are summarised.

### Study characteristics

Study design, participant and physiotherapy treatment characteristics are outlined for each study in Table 1.

**Table 1:** Study characteristics (divided into 3 sections according to study design)

**Study design**	**Author, date and country of clinical setting**	**Participants: Start (S) n = Finish (F) n = Mean Age (A) and SD (range) years; Duration of Sh P (D)**	**Clinical diagnosis**	**Primary outcome measures**	**Follow up period**	**Loss to follow up (%)**	**PT treatment**	**Durat’nof PT course**	**No. of PT appoint-ments**	**Durat’n of PT appoint-ments**	**Proport’n attend-ing ≥ 1 session of PT (%)**	**Proport’n attending full PT course (%)**	**Compliance with home exercises (%)**
Controlled Trial (CT), 2 groups diff. @ start receive same PT	Aydogan 2003/2004 [24] Turkey	S:n = 48 F:n = NS A:60 ± 7 & 58 ± 10 (44–80) D:≥3 m	Adhesive Capsulitis	Pain, active and passive ROM, Constant Score	i) On completion of PT course at 4 wks, ii) 3 m after D/C	UTD	Home exs, Stretches (Home and physiotherapist applied), Pulsed ultrasound, TNS	4w, 3 m HEP	20	UTD	UTD	UTD	UTD
CT, 2 groups diff. @ start receive same PT	Kim 2004 [17] South Korea	S:n = 90 F:n = 81 A:PLJG 24(19–31),PFJG 25(18–29) D:PLJG 4 ± 4,PFJG 3 ± 4y	Posterior Inferior Instability	UCLA, ASES, ROWE, Pain	6 m (range 4–7)	10%	Supervised and home exercises	6 m (range 4–7)	UTD	UTD	100	90	70
RCT, 2 groups similar @ start receive different PT	Conroy 1998 [22] USA	S:n = 7 + 7, F:n = UTD A:51(17)&55(10) D:NS	Shoulder Impingement Syndrome (Primary)	Pain, AROM sh Fl, Abd, IR, ER & scap. plane El, pain, functional Ax	1-3 d after completion of PT course at 3 wks	UTD	Supervised/HEP, advise, heat, massage, DTFs, soft tissue mobs, 1 group received "Maitland" mobs to sh.	3 w	9	UTD, (Incl exs 45-60mins, hotpacks 15 mins)	UTD	96	82
RCT, 2 groups similar at start receive different PT	Engebretsen 2010 [23] Norway	S:n = 104 F:n = 102; A:48 ± 11y D:3-6 m 33%, 6-12 m 29%, >12 m 39%	Sub-acromial Impingement Syndrome	SPADI; working/not working	1 year	10%-12%	1) Advice and supervised exercises or 2) extracorporeal shockwave therapy.	Gp 1) ≤ 12 w Gp 2) 4–6 w	1) Median 15 (IQR 11–16) 2) median 5 (IQR 4–6)	Gp 1) 45 m Gp 2) NS	≥98	90-96	UTD
RCT, 3 gps similar @ start receive different PT	Tanaka 2010 [18] Japan	S:n = 120 F:n = 110 A:64 ± 9y D:<1 m 34%, <3 m 35%, <6 m 19%, <7 m 12%	Adhesive Capsulitis	Change in active range of shoulder abduction	When improvement in O/C plateaued > 1 m (mean 5.9 ± 1.3 m)	8%	Manual therapy to the shoulder, home exercises	5 ± 1 m	3 groups i) >2× w ii) 1× w iii) < 1× w	40 mins	UTD	UTD	53
Cohort	Deutscher 2009 [31] Israel	S:n = NS, F:n = 5252 A:56 ± 15 D :0-21d 15% 22-90d 38% >90d 47%	NS	Functional Status using CAT [40]	On completion of PT course	~61% of full pop'n	At PT's discretion: advice, US, heat/ice, DTF, home/sup'd/class exercises, electrotherapy, MT, sh jt or soft tissue mobs	8 ± 6w	9 ± 6	26 ± 8 mins	95-100	98	70 good, 14 mod
Cohort	Griggs 2000 [25] USA	S:n = 75, F:n = 71 A:53(35–76) D:9(1–47)m	Adhesive Capsulitis (idiopathic phase II, P & limited ROM)	DASH, SF-36, Pain, active and passive ROM and SST	At i) 6–12 ws ii) 22 (12–41)m	4% at 22 m	Home exs, 68 (91%) patients participated in supervised exercise programme	UTD	NS	UTD	91	UTD	mean 2× of recomm-ended 5 × daily
Cohort	Hung 2010 [20] Taiwan	S:n = 33 F:n = 32; A:20-33y, D:“improvers” 23 ± 18 m,“non improvers” 29 ± 15 m	Sub-acromial Impingement Syndrome	GROC	On completion of PT course at 6 wks	3%, n = 1	Exercises, manual therapy to shoulder & patient applied stretches	6 wks	≤12	UTD	97	>80	UTD
Cohort	Kennedy 2006 [32] Canada	S:n = 361 F:289 A:50 ± 15 D: <4w 24%, 4-12w 25%, >12w 49%, missing 3%	Actively not sub-classified. Soft tissue, pain/dis-comfort, 8% post surgery.	DASH	Max 12 weeks or discharge from PT if earlier	20%	UTD	≤12 wks	Mean 15 (±9)	UTD	100	UTD	UTD
Cohort	Mao 1997 [26] Taiwan	S:n = 18 → 12 F:n = 12 A:52(32–65) D:2-12 m	Adhesive Capsulitis	Range of movement (?active or passive)	After PT	UTD	Supervised and home exs, manual therapy to shoulder, electrotherapy	4-6 wks	2-3 × a week (8 to 18)	UTD	100	UTD	UTD
Cohort	Mintken 2010 [21,69] USA	S:n = 80 F:n = 79 A:41 ± 13y D:511 ± 1503d	Mechanical Shoulder Pain	GROC, days off work 48 hrs after initial PT	2nd or 3rd appointment over several days	<1%	MT to cervicothoracic spine and spinal mob'g exercises	<2 wks	2 or 3	Techniques <15 mins	100	99	UTD
Cohort	Ryall 2007 [16] UK	S:n = 165 F:132 A:15–44 29%, 45–54 33%, 55–64 38%	Actively not sub-classified	Pain	1, 3, 6 & 12 months	20%	Physio’s discretion	UTD	UTD	UTD	UTD	UTD	UTD
Cohort	Sindhu 2012 [29] USA	S:n = 3362 F:n = 1946-1519 A:54 ± 16y D: <22d 19% 22-90d 32%,% < 90d 49%	Musculo-skeletal conditions of the shoulder	Functional status using CAT [40] & Pain	On discharge from PT	43%-53%	UTD	UTD	UTD	UTD	UTD	UTD	NS
Cohort	Tyler 2010 [28] USA	S:n = 22 F:n = 22 A:41 ± 13y D:5 ± 5 (1-24mo)	Posterior Impingement	Simple shoulder test	On discharge	0%	MT to shoulder and home exercises	7 ± 2 wks (3–12)	UTD	UTD	100	UTD	UTD
Cohort	Virta 2009 [27] Norway	S:n = 97 F:n = 72 A:50, median 51 (24–80) D:1-36mo	Shoulder Impingement Syndrome	UCLA	UTD	26%	Supervised and PT facilitated exercises and home exercises	Mean 8 wks	Mean 11	1 hr	UTD	74	UTD
Cohort	Yang 2008 [19] Taiwan	S:n = 40 F:n = 34 A:54 ± 6(41–65)y D:6 ± 8 m (range 3–9 m)	Adhesive Capsulitis	FLEX-SF	3 months	15%	MT to shoulder, electrotherapy, supervised exes and PT app’d stretches	3 mo	~24, (2× w)	UTD	100	85	NA

Legend: SD standard deviation, Durat’n duration, Proport’n proportion, NS Not stated, years, m months, w weeks, hr hour, d days, mins minutes, UTD unable to determine, ROM range of movement, AROM active range of movement, jt joint, PT physiotherapy, HEP Home exercise programme, DTF deep transverse frictions, MT Manual therapy, US Ultrasound, D/C Discharge, PLJG Painless jerk group, PFJG Painful jerk group, sh shoulder, Fl flexion, Abd abduction, Scap scapula, El Elevation, exs exercises, popn population, sup’d supervised.

#### Study design

Of the 16 studies finally selected for the review, eleven were cohort studies and five were controlled trials. Three of the controlled trials randomized participants into 2 or more groups, all of whom received some form of physiotherapy [18,22,23]; two divided participants into two groups according to differences in baseline characteristics and administered the same physiotherapy treatment to both groups [17,24].

#### Classification of shoulder pain

Clinical eligibility criteria were provided in enough detail to allow transferability of findings to clinical practice in 11 of the 16 studies. However a common omission was clarification that somatic referred pain from the cervical spine, distinct from radiculopathy, was excluded as a source of shoulder pain; one study [21] excluded patients with nerve root signs and another excluded patients with cervical spondylosis [24], three studies [19,22,23] stated that the cervical spine was excluded as a source of referral, but only one study [22] stated the mechanism by which this decision was made. One study purposely did not exclude participants with cervical spine pathology [25]. Five studies only included participants with adhesive capsulitis [18,19,24-26], four studies only included participants with subacromial impingement syndrome [20,22,23,27], one study only included participants with posterior inferior instability of the shoulder [17] and one study only included participants with a positive posterior impingement sign and the presence of a posterosuperior glenoid labral lesion on MRI [28]. One study [29] used the International Classification of Diseases (ICD-9) codes [30] to divide “musculoskeletal shoulder pain” into 8 disease categories. The authors themselves report ICD-9 codes as lacking specificity and reliability, yet rather than report comprehensive results for their full cohort, only report results for these disease specific categories. Within each sub-group of shoulder classification, no two studies used the same eligibility criteria. Five studies, [16,21,29,31,32] did not sub-categorize shoulder pain using a clinical diagnosis; all providing minimal details of eligibility criteria. However these results are transferable to the wider range of patients.

#### Physiotherapy treatment

The number of participants receiving physiotherapy treatment ranged from 14 [22] to 5252 [31]. Of the 13 (of the total of 16) studies that reported any details of physiotherapy, treatment included home exercises (n = 10), supervised exercises (n = 9), exercises (unable to determine whether supervised or at home, n = 2), manual therapy to the shoulder (n = 7), treatment applied to the spine (n = 1) and electrotherapy (n = 4). Prognostic factors and outcomes varied across studies.

### Quality assessment of external validity, risk of bias, and presentation of results

The assessment of study quality based on the 24 items (Additional file 3) is presented in Table 2. Over two thirds of studies identified a priori and reported baseline prognostic factors and outcome measures using standardized measurement tools, and reported percentage loss to follow up. None of the studies stated whether outcome assessors, including participants completing patient rated questionnaires, were blind to baseline prognostic variables.

**Table 2:** Quality assessment of external validity, risk of bias, and presentation of results

**Section**	**Assessment criteria**	**A**	**B**	**C**	**D**	**E**	**F**	**G**	**H**	**J**	**K**	**L**	**M**	**N**	**0**	**P**	**Q**	**R**	**S**	**T**	**U**	**V**	**W**	**X**	**Y**	**Total**
1	Aydogan 2003/2004 [24]	0	1	0	0	1	0	1	1	1	1	0	0	0	0	0	0	0	0	0	0	0	0	0	0	6
	Kim 2004 [17]	0.5	1	0	1	1	0	1	1	1	1	1	1	1	0	1	1	0	0.5	1	0	1	1	0	0	15.5
2	Conroy 1998 [22]	0	1	0	1	1	0	1	1	1	1	0	1	1	0	1	0	0	0	0	0	0	0	0	0	10
	Engebretsen 2010 [23]	0	1	0	1	1	0	1	1	1	1	1	1	0	0	0	1	1	1	1	1	1	1	1	1	18
	Tanaka 2000 [18]	1	0	0	1	1	0	1	1	1	1	0	0	1	1	0	1	0	1	1	0	0	0	1	1	14
3	Deutscher 2009 [31]	1	0	0.5	1	1	1	0	1	1	1	1	1	1	0	1	0.5	1	0	0	0	0	1	1	0	16
	Griggs 2000 [25]	0	1	0	0	1	0	0	0	0	0	1	0	1	0	0	1	0	0	1	0	0	0	0	0	6
	Hung 2010 [20]	0	0.5	0	1	1	0	1	1	1	1	1	1	0	0	1	1	1	0.5	1	0	0	1	1	1	16
	Kennedy 2006 [32]	1	0.5	0.5	0.5	1	0	1	1	1	1	1	0	0	0	0	1	0	1	1	0	0.5	1	1	1	14
	Mao 1997 [26]	0	0.5	0	1	0	0	1	1	1	1	1	0	0	0	0	0	0	0.5	0	0	0	0	0	1	8
	Mintken 2010 [21,69]	1	0	0	1	1	0	1	1	1	1	1	1	0	0	1	1	1	1	1	1	0	1	1	1	18
	Ryall 2007 [16]	1	0	0	1	1	0	1	1	1	1	0	0	0	0	0	1	0.5	1	1	0	0	1	1	1	13.5
	Sindhu 2012 [29]	1	0	0.5	1	1	1	0	0	1	1	1	0	0	0	1	1	1	0.5	0	0	0	0	0	0	11
	Tyler 2010 [28]	0	0.5	0	1	1	0.5	0	0	1	1	1	0	0	0	0	1	1	0	1	0	0	0	0	0	9
	Virta 2009 [27]	0	0.5	0	1	1	0	1	1	1	1	0	1	0	0	0	1	0	0	0	0	0	0	0	0	8.5
	Yang 2008 [19]	0	1	0	1	1	0	1	1	1	1	1	1	1	0	0	1	0	0.5	1	0	0	1	1	1	15.5

Section 1: Two groups, different at baseline, receive the same treatment; Section 2: Randomised controlled trials; Section 3: Cohort studies.

0 = No or unable to determine, 1 = Yes or not applicable, 0.5 = In part, to an extent that neither 0 or 1 applicable.

#### Population representation at baseline

Proportional eligibility was often stated but only four studies explicitly reported recruitment rate in proportion to those eligible and/or invited onto the study [16,18,23,32]. Research investigating areas other than shoulder pain have identified differences in baseline characteristics between potential participants who consent and do not consent [33-35]. One study [32] within this review compared demographic variables between participants and non-participants and found no difference between groups with respect to age and sex, although non-participants had a longer duration of symptoms than participants (381 v 229 days, p = 0.07). Generally baseline information for potential participants who do not consent is by definition restricted, making comparisons at best limited.

#### Appointment attendance and exercise compliance

There is evidence that treatment adherence is correlated with a better treatment outcome [36,37]. The number of participants not completing the full course of physiotherapy was either not stated or below 80% in nine of the 16 studies. One study [31] within this review investigated and demonstrated an association between good appointment attendance and better outcome (n = 5252, p < 0.001). Home exercises were prescribed in ten studies; six reported rates of compliance [17-19,22,25,31]. Two studies within this review investigated the association between home exercise compliance and outcome. Deutscher [31] demonstrated that good home exercise compliance was the joint second most predictive variable for a better outcome (p < 0.001). Tanaka [18] demonstrated a significant improvement in range of abduction and over a shorter time period for participants performing their home exercises daily in comparison to those not doing them at all (p = <0.001). A shorter time period to full improvement was also demonstrated for those who exercised daily in comparison to several times a week (P < 0.017). Tanaka was the only study to explicitly state whether participants received additional treatment to the package defined at onset [18]. Appointment attendance and compliance with home exercises should be recorded and analyzed as possible interactions when investigating the correlation between baseline prognostic factors and treatment outcome.

#### Presentation of results

Presentation of results varied considerably. Only two studies [28,29] included a power analysis, one of which was retrospective [29]. Some studies included within the review may have suffered from a type I or type II error. A number of studies demonstrated clear trends between some prognostic factors and outcome which were not statistically significant. Seven studies [22,24-29] omitted details of random variability and measures of association between prognostic variables and outcome (or differences between prognostic groups). The material available to present in our results section for these studies was therefore minimal. In addition four of these studies did not report precise p-values so that the probability of any association (or differences between prognostic groups) being due to chance was not available if more than 5% [22,24,27,29]. None of these studies reported on more than three of nine items assessed within our quality criteria specifically for reporting results. With two exceptions [22,29], these same studies did not report on more than half the items within our quality assessment criteria specifically selected for external validity and potential for bias (Table 1 and Additional file 3). The limited results described within these seven studies were therefore not reported. The remaining nine studies met between 13.5 and 18 of the 24 criteria. Within the relevant subheadings, studies meeting the highest number of quality criteria are presented first.

#### Loss to follow up

It was important to ascertain whether participants lost to follow up were a random subset of the whole or if there was a systematic difference between groups which if ignored may affect outcome [38]. In 12 of 16 studies for which it was reported, loss to follow up ranged from 0% to approximately 61%. Patients who completed and did not complete final follow up were compared on baseline characteristics in four studies [16,23,29,31]. Two of these studies only included participants who had completed physiotherapy and provided discharge data; this was from a larger group of patients attending physiotherapy whose details had been captured on an electronic database at the start of physiotherapy [29,31]. The high loss to follow up in these two studies is probably reflective of this mechanism of participant selection. The 43-53% (depending on outcome) of patients in Sindhu et al’s study who did not complete physiotherapy and/or were lost to follow up at discharge, and therefore not selected for the study, were significantly different (p < 0.05) from those completing physiotherapy and available for follow up at discharge [29]. This was in terms of age, geographic region, pain intensity and function at intake; directional details are not provided. Patients not completing physiotherapy and lost to follow up for a range of conditions in addition to the shoulder in Deutscher et al’s [31] study (n was approximately 61%) were more likely to have a history of 90 or more days of pain and more co-morbidities than those completing physiotherapy and not lost to follow up (p < 0.001). Of the 10 participants lost to follow up in Engebretson et al’s study [23], 80% (n = 8) were not working at baseline in comparison with 25% (n = 24) who completed the one year follow up. Participants lost to follow up were slightly older (57 versus 49 years) and had a higher mean SPADI score at baseline (56 versus 49) compared with the study group as a whole. Ryall [16] reported no significant differences (p > 0.05) in age, gender, somatising tendency, and scores for anxiety, depression, hypochondriasis and health beliefs for those available and not available to follow up. How much the results of these studies reflect the profile of participants lost to follow up in similar studies cannot be gauged.

### Results from individual studies

Prognostic factors were reported for six different outcome categories. A number of individual studies investigated over 15 prognostic factors [16,21,23,31,32]. For each outcome measure, results of studies meeting the highest number of quality assessment criteria will be presented first. Predictive factors found to have a significant association with any of these outcomes (on multiple regression analysis or equivalent) in two or more of the studies will be summarised in the section following.

#### Patient-rated functional outcome

Five of the nine studies within this review for which results for patient rated functional outcomes are reported, used a total of seven different questionnaires, none of which were used by more than one study.

One study, meeting 18 of our 24 quality assessment criteria, investigated the potential predictive factor of approximately 16 baseline characteristics on the shoulder pain and disability index (SPADI) [39] at one year follow up [23]. Univariate linear regression identified 11 possible predictors (p < 0.1), only three of which were retained in the final backward multiple regression model (Additional file 4). Lower education, previous shoulder pain and high baseline SPADI predicted poor outcome and accounted for 30% of the variance in the final SPADI score at one year.

One study [31] meeting 16 of our quality assessment criteria investigated the association of approximately 22 baseline characteristics with the computerized adaptive test (FT-CAT) [40] at discharge. Only statistically significant results were presented in their multiple regression analysis (Additional file 5). In this table ß is “*the coefficient that represents the amount of expected change in discharge* [FT-CAT] *given a 1-unit change in the value of the variable, given that all other variables in the model are held constant*” [31]. These factors accounted for 30% of the variance in FT-CAT at discharge; a negative beta value (ß) is associated with a poor outcome and a positive beta value (ß) is associated with a better outcome.

One study [17] meeting 15.5 of our quality assessment criteria divided participants with posterior inferior instability of the shoulder (n = 81) into those with (n = 33) and without a painful jerk test (n = 48) [41]. This test involves stabilising the scapula and concurrently applying an axial force along the humerus whilst the shoulder is placed in 90 degrees abduction and internally rotated. The arm is then horizontally adducted whilst maintaining the axial load. A clunk is indicative of the humeral head sliding off the back of the glenoid and is the criteria for a positive test, a second clunk may be observed as the arm is returned the start position and the humeral head relocates [17]. Clinically and statistically significant improvements (Mann–Whitney *U* test, p < 0.001) were demonstrated in functional status measured by the i) Rowe Score for Instability [42], ii) University of California-Los Angeles Shoulder Scale (UCLA) [43] and iii) modified American Shoulder and Elbow Surgeons Shoulder Index (ASES) [44] for participants with a painless compared with a painful jerk test following a 6 month rehabilitation programme (Additional file 6).

Another study [19] meeting 15.5 of our quality assessment criteria divided participants into improvers and non-improvers based upon a positive change of more than 20% on the Flexilevel Scale of Shoulder Function (FLEX-SF) [45] over a 3 month rehabilitation period. Two of three movements of the shoulder complex, detectable by clinical examination (rather than laboratory testing) were significantly different between groups and were included within a clinical prediction model; humeral elevation > 97° and external rotation > 39° at baseline were associated with successful treatment (Additional file 7).

One study [32] meeting 14 of our quality assessment criteria investigated the association between approximately 24 baseline characteristics and i) final Disability of the Arm, Shoulder and Hand (DASH) scores [46] and ii) change in DASH scores 12 weeks after commencing physiotherapy. Twenty one factors were significant (<10% probability of chance) on univariate analysis and advanced to the final multiple regression models (Additional file 8). There is some inconsistency of reporting; the authors’ narrative summary states that being female is a predictor of greater disability at discharge, yet the statistical presentation of results suggests the opposite; that being female is a predictor of lower DASH score (i.e. better function) at discharge. Similarly higher pain intensity and previous shoulder surgery appear to be statistically predictive of deterioration yet are reported as predictors of improvement. Only one of five predictive factors, younger age, was common to both outcomes, and predicted a better outcome. This highlights that seemingly similar outcomes can have associations with very different predictive factors [32].

#### Global impression of change (GROC)

Two studies meeting 18 [21] and 16 [20] of our quality assessment criteria investigated whether treatment success, based on a score of +4 on the 15 point Patient Global Rating of Change (GROC) [47] was associated with approximately 27 and approximately 12 baseline measures respectively. Following logistic regression analysis, Mintken [21] included five factors within a clinical prediction rule developed to identify the patients most likely to improve after 1–2 treatments of cervico-thoracic manipulation. Three of these factors (duration of shoulder pain, range of shoulder flexion and internal rotation) were also investigated in a smaller study by Hung [20]; no association with successful treatment (P ≥ 0.3) was demonstrated (Additional file 9). Hung [20] associated successful treatment with reduced strength of the humeral external rotators (p = 0.076), serratus anterior (p = 0.040) and lower function, indicated by lower FLEX-SF scores [45] (p < 0.00005) at baseline. In their final model only the latter two were included together with an additional measurement from laboratory testing. These factors were not investigated by Mintken [21].

#### Pain

Two studies [16,17] meeting 15.5 and 13.5 of our quality assessment criteria investigated the association of potential predictive factors with the outcome of shoulder pain following physiotherapy treatment. Kim et al. [17] demonstrated that the group of participants with a painless rather than painful jerk test [41] had significantly lower mean pain scores at follow up (Mann–Whitney *U* test, p < 0.001) (Additional file 10). Ryall et al. [16] investigated the potential predictive factor of approximately 17 baseline characteristics on the prevalence of three aspects of “same site pain” at 12 month follow up. Whilst the odds of continuing pain in terms of point prevalence was higher for a number of baseline characteristics, with only two exceptions, (Additional file 10), confidence intervals passed through one. The lack of statistical significance may reflect the lower power of this sub group analysis specifically undertaken for this review [Personal communications: Palmer K and Ntani G, University of Southampton, 2012].

#### Work

Two studies, both of which met 18 of our 24 quality assessment criteria, investigated baseline characteristics as predictors of whether or not participants were working either 48 hours after the first physiotherapy treatment [21] or at one year follow up [23] (Additional file 11). Within the 48 hour treatment period, high fear avoidance beliefs specific to work, measured by the Fear Avoidance Beliefs Questionnaire – Work Beliefs [48] were strongly predictive of missing work, although lower scores were not predictive of remaining at work [21]. Fear avoidance specific to physical activity, measured by the Fear Avoidance Beliefs Questionnaire – Physical Activity [48] was not associated with outcome. At one year follow up Engebreston [23] identified a number of possible predictors on univariate linear regression, only two of which were included in the final forward logistic regression model. Higher education and better self-reported health status were predictive of working at one year.

#### Range of movement

One study meeting 14 of our quality assessment criteria investigated the potential predictive factor of four baseline characteristics on i) improved range of active abduction and ii) point in time at which improved range had plateaued for more than one month, in 120 participants with adhesive capsulitis [18]. Statistically significant predictors of improved range of abduction included younger age, shorter duration of symptoms and hand dominance (Additional file 12). No difference between categories was detected in time for improvement to plateau.

#### Adverse outcomes

Two studies reported adverse outcomes [21,23], one over a year [23] and the other over a maximum two week follow up [21,49]; the later treatment included spinal manipulation. No adverse events were observed. Four studies reported how many participants were worse [17,21,23,32,49] or remained the same [17] during treatment [23] or at follow up [17,21,32,49] (Additional file 13). Getting worse with physiotherapy was clearly related to a painful jerk test in Kim et al’s study [17]. However in the few studies for which it was reported, less than 10 per cent of participants worsened with physiotherapy.

### Summary of results

Some predictive factors were found to have a significant association with outcome from physiotherapy treatment (on multiple regression analysis or equivalent) in two or more of the studies described above. For these predictive factors, the results are synthesised and summarised below.

#### Function at baseline

Three studies investigated the association of functional disability at baseline with functional outcome. Results were consistently significant in the same direction on multiple regression analysis; high baseline disability was associated with poor functional outcome [23,32], low baseline disability was associated with a better functional outcome [31]. Two studies investigated the association of baseline disability with successful treatment defined by the global rating of change (GROC). Results were inconsistent; one study did not detect any difference between successful and unsuccessful treatment groups [21], the other associated higher baseline disability with better outcome [20].

#### Duration of shoulder symptoms

Six studies investigated the association between duration of shoulder symptoms and outcome. Longer duration of symptoms was consistently associated with a poorer outcome [18,31] and shorter duration of symptoms with a better outcome [21,31,32]. Engebretson demonstrated a similar pattern on uni-variate but not multi regression analysis [23] and although statistically insignificant, visual inspection of Hung et al’s [20] results indicates a similar trend. The latter were the two studies reported within this review which only included participants with subacromial impingement syndrome.

#### Age

Six studies investigated the association between age and outcome. Two studies demonstrated an association between increasing age and poorer functional outcome on multiple regression analysis [31,32] and one study demonstrated that older age groups experienced less improvement in range of shoulder abduction [18]. No association between age and outcome was demonstrated in the remaining three studies [16,20,23].

#### Range of shoulder flexion

The association between baseline range of movement and outcome was less consistent. Two studies identified range of shoulder flexion at baseline as a predictor of outcome; one study demonstrated that greater restriction of flexion was predictive of a good outcome (GROC) [21], the other demonstrated that less restriction of flexion was predictive of a better functional outcome [19]. Two studies identified an association on uni-variate but not multivariate analysis [23,32] and one study reported no association [20]. The latter included the two studies reported within this review which only included participants with subacromial impingement syndrome.

## Discussion

There was consistent evidence from two or more studies meeting 13 or more of our 24 quality assessment criteria, of an association between the following predictive factors and outcome i) higher disability at baseline was predictive of a higher disability at follow up or low disability at baseline was associated with a lower disability at follow up ii) longer duration of shoulder symptoms was associated with poorer outcome or shorter duration of symptoms with better outcome, iii) increasing age was associated with poorer outcome. Restricted range of shoulder flexion predicted outcome in two studies; however one study demonstrated that higher shoulder flexion at baseline (>97°) was predictive of a good outcome and another demonstrated that lower shoulder flexion at baseline (<127°) was predictive of a good outcome.

For many potential prognostic factors results were inconsistent between studies. Clinical heterogeneity in terms of the presentation of shoulder pain, treatment type, dose, duration, attendance, compliance, as well as differences in follow up period and measurement tools may account for some of the variability of results and their significance. Physiotherapy attendance rates and adherence to prescribed exercise is important as this review seeks to identify prognostic factors specific to physiotherapy treatment rather than simply referral to physiotherapy and for non-attenders, the natural course of shoulder pain.

Patients present to physiotherapy with shoulder pain arising from a number of potential sources. Studies which included patients with upper quadrant pain but did not clearly state the shoulder as the source of symptoms were excluded from this review. However eligibility criteria differed considerably between studies and it was not always clear that the cervical spine was explicitly cleared as a potential source of symptoms. Based on the patient’s history and physiotherapist’s clinical examination shoulder pain is sometimes categorised using a number of diagnostic labels. Studies which sub categorise shoulder pain may detect prognostic factors which may not be detected in a more generic patient group.

Within this review two sub-groups of shoulder pain contained more than one study; adhesive capsulitis and subacromial impingement syndrome. Within these subgroups, no two studies used the same eligibility criteria. This lack of standardisation or discrepancy in labelling shoulder pain has been reported previously [50-52]. Differing exclusion as well as inclusion criteria can contribute to heterogeneity between studies seemingly investigating the same subgroup of patients with shoulder pain and hamper effective comparison [50,51].

Meaningful sub group analysis according to any criteria was limited by heterogeneity in other areas. Studies within both the adhesive capsulitis and subacromial impingement syndrome groups used different outcome measures. In addition the two studies for which results were reported for participants with adhesive capsulitis investigated different prognostic factors [18,19], rendering comparisons impossible. On final multivariate analysis duration of symptoms and range of shoulder flexion did not demonstrate any statistical association with outcome for the two studies reporting results for participants with subacromial impingement syndrome [20,23]. However a trend was observed between duration of symptoms and outcome in these latter two studies and reflects the findings of the review overall, and the findings in the three studies [16,31,32] reported, which included participants with a variety of shoulder presentations including subacromial impingement.

There is evidence of poor inter-rater reliability for the sub-classification of shoulder pain [53,54]. As stated previously the majority of studies within this review clearly outlined their eligibility criteria. However earlier reviews have demonstrated that most clinical tests used for the sub-classification of shoulder pain demonstrate poor diagnostic accuracy [55,56]. A number of studies used radiological findings as eligibility criteria [17,18,24,25,27,28]. However in the physiotherapy clinic radiological findings are not always clinically indicated and if present, details may not be accessible to physiotherapists at the first appointment. In addition there is often a poor correlation between structural pathology and the clinical presentation of shoulder pain [57-63]. Some researchers have suggested that musculoskeletal shoulder pain should not be sub-categorised according to structural pathology [50,52,64]. The four largest studies in this review did not sub-categorise shoulder pain [16,29,31,32], two stating that this was an active decision based upon the poor reliability of shoulder classification [16,32].

In the field of musculoskeletal low back pain many clinicians base decision making about initial management options based upon prognostic indicators [65] rather than diagnostic classifications. This in part builds upon similar observations for the poor reliability of structural diagnoses and their poor correlation with clinical presentation [66].

To our knowledge, this is the first systematic review of the current literature on potential predictive factors specific to the outcome of physiotherapy management for musculoskeletal shoulder pain. A previous systematic review of cohort studies investigated potential prognostic factors irrespective of management type. Two of their 16 studies included physiotherapy management; these were excluded from our review because they were retrospective analyses [67,68]. Overall their review reported strong evidence that aged 45–54 years in occupational settings and high pain intensity in primary care were strong predictors of a poor prognosis. Age was not a factor considered in relation to work status for the studies in our review. However one [32] of two [20,32] studies demonstrated a strong correlation between high pain intensity at baseline and poor prognosis. Within a primary care setting these same researchers reported some evidence that longer duration of shoulder symptoms and high disability at baseline were predictors of poor prognosis. These were the strongest predictors within our review of outcome specific to physiotherapy treatment.

### Potential biases in the review process

The main search for this review was restricted to four databases. Studies presenting interesting or significant findings are more likely to be published than those with non-significant findings [33]. Conference proceedings per se were not included within our search, however there is evidence that higher quality conference abstracts are more likely to be published as a full article than lower quality abstracts [34] and the inclusion of unpublished potentially poorer quality material in reviews may actually be a source of bias [35]. Searching the grey literature is important when the objective is avoidance of biasing review results towards significant reports of prognostic factors. However this review is the first of its kind and our intention was to gather evidence of the most likely significant predictive factors of outcome for further investigation. Studies were therefore required which were presented in enough detail to carry out a quality assessment appraisal and of a standard appropriate for peer reviewed publication.

Although a number of validated quality assessment tools are available, none covered all the criteria important for a study addressing our objectives. Criteria were therefore selected from a number of sources. Whilst our method of quality assessment was repeatable within our team, reproducibility has not been tested externally and the number of criteria met should not be confused with a scoring system. Seven studies provided minimum reporting of results specific to our objective and were therefore omitted from our results section due to the quality of reporting but also on a pragmatic basis.

#### Implications for future research

Large adequately powered prospective studies are required and should include as a minimum, investigation of the association between baseline disability, age, duration of shoulder symptoms and range of shoulder flexion with functional outcome. Inclusion of the additional significant prognostic factors identified on multiple regression analysis or equivalent by the nine studies presented within this review should also be considered given the possibility of a type II error in some of these studies. Eligibility criteria should apply to somatic as well as radicular referral of pain from the cervical spine as the primary source of symptoms. Given the common omission of any detail of concurrent pain from other sources in the affected upper quadrant, it would be appropriate to include this as a possible predictive factor. Given the poor reliability of shoulder classification systems based on diagnostic labels and the poor correlation between structural pathology and clinical presentation, eligibility criteria should be based upon patient characteristics and reproducible baseline data. Exercise adherence, treatment attendance and whether or not participants have completed the full course physiotherapy should be recorded as these have been demonstrated to have a significant effect on treatment outcome. Comparisons should be made between participants available and not available for follow up and the results should inform the final analysis. Information about patients who are eligible and have been invited to take part in a study but have not consented will be absent or limited at best. However the proportion of eligible patients who were asked and agreed compared to those who did not agree to take part should be stated and for those factors on which data may be available, differences stated. As well as predictors for participants who will improve with physiotherapy, analysis should include predictors for those whose shoulder symptoms may worsen during physiotherapy.

## Conclusion

Associations between prognostic factors and outcome were often inconsistent between studies. This may be reflective of a type II error or heterogeneity on a number of levels including treatment selection, adherence or outcome measure. Only two baseline prognostic factors consistently demonstrated anassociation with outcome in two or more studies; duration of shoulder pain and baseline function.

Decisions based on prognostic factors may be clinically more useful given the poor reliability of shoulder sub-categorization based on diagnostic labels. Prior to developing a predictive model for the outcome of physiotherapy treatment for shoulder pain, a large adequately powered cohort study is required in which a broad range of prognostic factors are incorporated.

## Competing interests

The authors declare that they have no competing interests.

## Authors’ contributions

RC co-coordinated the review, carried out the literature search, data analysis and drafting of the manuscript and contributed to study selection, data extraction, and quality assessment of included studies. DS and HD contributed to the study selection, data extraction, quality assessment of included studies and critically revised the manuscript. JL provided expert clinic advice in the field, quality assessment of included studies and critically revised the manuscript. LS provided expert advice on design and statistics, contributed to data analysis and critically revised the manuscript. CJH provided expert advice on systematic reviews, design and structure and critically revised the manuscript. All authors read and approved the final manuscript.

## Pre-publication history

The pre-publication history for this paper can be accessed here:

http://www.biomedcentral.com/1471-2474/14/203/prepub

## Supplementary Material

Additional file 1 Search strategy used in MEDLINE. Click here for file

Additional file 2 Data Extraction Form. Click here for file

Additional file 3 Criteria for the assessment of external validity, risk of bias, and presentation of results specific to the objectives of this review. Click here for file

Additional file 4 **Engebretson et al’s **[23]** multiple regression model (backward) predicting SPADI at one year follow up and synopsis of uni-variate analysis (n = 104).** Click here for file

Additional file 5 **Deutscher et al’s **[31]** multiple regression analyses predicting functional status, (CAT) **[43]** at discharge.** Click here for file

Additional file 6 **Kim et al’s **[17]** functional outcomes for groups with and without a painful jerk test **[41]**.** Click here for file

Additional file 7 **Accuracy statistics for the factors retained within Yang et al’s **[19]** clinical predication rule (for a positive outcome).** Click here for file

Additional file 8 **Kennedy’s **[32]** multiple regression models (n = 289) for DASH scores at discharge or 12 weeks and synopsis of uni-variate analysis.** Click here for file

Additional file 9 **Accuracy statistics for the factors retained (and synopsis of those excluded) within Mintken et al’s **[21]** and Hung et al’s **[20]** clinical predication rules (for a positive outcome).** Click here for file

Additional file 10 **Predictive Factors for Pain: Kim et al’s **[17]** and Ryall **[16]** [Personal communications: Unpublished data.** Palmer K and Ntani G, University of Southampton, 2012]. Odds ratios and 95% confidence intervals for statistically significant factors (p≤0.5) and synopsis of results not reach significance (p>0.5). Click here for file

Additional file 11 **Predictors for time off work, Engebretson’s **[23]** logistic regression model (forwards) and Mintken’s **[21,69]** predictive statistics.** Click here for file

Additional file 12 **Tanaka’s **[18]** comparison of outcome (improvement in abduction) for each category.** Click here for file

Additional file 13 **Studies **[17,21,23,32,69]** (n = 4) reporting numbers who get worse during physiotherapy or at follow up.** Click here for file
