# Supplementary material for: Predicting response to physiotherapy treatment for musculoskeletal shoulder pain: a systematic review
Source: BMC Musculoskelet Disord. 2013 Jul 8;14:203. doi: 10.1186/1471-2474-14-203 (PMC3717132; doi:10.1186/1471-2474-14-203)
Supplement: Additional file 1 — Search strategy used in MEDLINE. [file 1471-2474-14-203-S1.pdf]

Additional File 1: Search strategy used in MEDLINE

MEDLINE

Condition

MESH terms: Shoulder pain/ or Shoulder Impingement Syndrome or Rotator Cuff/ or exp Bursitis

Text words: ((shoulder$ or rotator cuff) adj5 (bursitis or frozen or impinge$ or instab$ or tendin$ or tendon$ or sublux$ or pain$)).mp

Adhesive capulitis.mp

Rotator cuff.mp

Treatment

MESH terms: exp Rehabilitation/ or exp Physical Therapy Modalities/ or exp Musculoskeletal Manipulations/ or exp Ultrasonography interventional/ or exp Acupuncture therapies/

*Text words: (*rehabilitat$ or physiotherap$ or physical therap$ or manual therap$ or exercis$ or ultrasound$ or ultrasonograph$ or TNS or TENS or electrotherap$ or inject$ or mobili$ or acupunctur$ or hydrotherap$) mp. [mp=title, original title, abstract, name of substance word, subject heading word]

Publication Type

MESH terms: exp cohort studies/ or epidemiologic studies/ or longitudinal studies/ or follow up studies/ or prospective studies/ or clinical trial/

*Text words: (*prognos$ or predict$ or cours$ or cohort$ or follow upS or prospective or longitudinal$ or random$)

Text words: (single or double) adj (blind or placebo); (clinical$) adj (stud$ or trial$)
