# Supplementary material for: Predicting response to physiotherapy treatment for musculoskeletal shoulder pain: a systematic review
Source: BMC Musculoskelet Disord. 2013 Jul 8;14:203. doi: 10.1186/1471-2474-14-203 (PMC3717132; doi:10.1186/1471-2474-14-203)
Supplement: Additional file 2 — Data Extraction Form. [file 1471-2474-14-203-S2.pdf]

Additional File 2: Data Extraction Form

Year	
1st Author	
Title	
Country(s) of publication	

INDEX

Page	Data
1	Study Design
2	Setting for Participant Selection and Collection of Baseline Variables/Selection Criteria
3	Selection Criteria
4	Participant Details
5	Physiotherapy Treatment and Compliance
6	Treatment from Non Physiotherapists
7	Outcome Measures
8	Numbers completing and Loss to Follow Up
9	Questions for specific study designs
10	Results (Separate Document)

STUDY DESIGN (Tick correct box)

Observational / longitudinal study	
(R)CT which carries out a subgroup analysis relating outcome in one Physiotherapy treatment group to baseline variables (i.e. treats part of study as one cohort)	
(R)CT in which 2 or more groups of subjects , the *same* at baseline, receive *different* physiotherapy treatments/packages	
(R)CT in which 2 or more groups of subjects, *different* at baseline, receive *different* physiotherapy treatment/package BUT each group is treated as an independent cohort.	
(R)CT in which 2 or more groups of subjects *, different* at baseline, receive the *same* physiotherapy treatment/package	
(R) CT in which there are 2 or more groups, not all of which receive physiotherapy, but which are grouped together as one cohort for baseline predictors	
Other (please describe)	

PARTICIPANT SELECTION AND METHOD OF COLLECTING BASELINE VARIABLES

SETTING for participant selection (s) assessment (a) and treatment (t)
(If different Indicate S, A and T for all groups involved in prognosis analysis)

Country (s) of data collection						
Not stated	District Hospital	University/ Teaching/ Regional Hospital	Primary care (circle one below)<br>GP / Cottage/ Community hospital / Not stated	Private<br>Sports elite	Leisure/Sports /recreational athletes	Other (please state)

Were all patients for whom prognostic factors were being studied, drawn from the same or comparable sources?

Not stated	No	Yes	Ambiguous (please state)

Method of selecting patients for the study

Not stated	Part of RCT	Consecutive	All defined cases	Convenience	Volunteer	Other (please state)

Source of baseline data/prognostic variables

Not stated	Structured assessment specifically for RCT or this study	Clearly defined standard operating procedures	Therapists questionnaire/ data extraction form	Patient Questionnaire	Patient records	Self report	Other (detail)

SELECTION CRITERIA

(INC if they were included, EXC if they were excluded, leave blank if not discussed. If *none* discussed, tick first box).

Specific Syndromes/Pathologies in this study

Not stated	Non specific shoulder pain	OA GHJ	Adhesive capsulitis/Frozen shoulder
Shoulder impingement syndrome	Rotator cuff – large/massive tears	Tendinopathy	Shoulder instability
Other (provide details)			

Who made the shoulder diagnosis?

Physiotherapist *assessing* Patient - part of standard care		Physiotherapist *treating* Patient - part of standard care	
Not stated	Independent Assessor	Orthopaedic Consultant	Other

Specific Details of Shoulder Inclusion/exclusion criteria

Did participants have pain on one or more active movements of the shoulder?	Yes (detail)			No	Not stated
Was the cervical spine excluded as a likely source of symptoms?	Yes, via history/subjective assessment	Yes, via clinical examination	Yes – but how is not stated	No	Not stated
Were inclusion criteria for shoulder pain stated?	Yes, via history/subjective assessment	Yes, via clinical examination	Yes – but how is not stated	Not stated	
Was diagnosis confirmed by radiographic findings?	Yes – for all participants using	Yes – for some but not all participants (no/%) using	Yes – but how is not stated	No	Not stated
Was a specific duration of shoulder symptoms required?	Yes ≥ 3 months	Yes (other duration – state)		No	Not stated
Were patients who had previously received Physiotherapy for the affected shoulder included?	Yes – providing not for this episode of pain	Yes - providing not in the last (state)		No	Not stated
	Yes – if unsuccessful	Other:			

<u>Inclusion/Exclusion criteria</u>

INC if they were included, EXC if they were excluded, leave blank if not discussed.
If *none* discussed, tick the first box. State timeframes if stated

None discussed	"Any co-morbidity"	Bilateral shoulder pain	Cervical radiculo-pathy	"Neck pain"	Diabetes<br>Type I<br>Type II
Previous surgery on affected shoulder	Fracture of affected shoulder complex	Dislocation of affected shoulder requiring secondary medical care	Other shoulder conditions (list)	Specific Systematic Rheumatologic conditions	Any Rheumatological condition
CRPS of upper limb on affected side	Mental health psychiatric disorders	Depression	MAJOR illnesses	Cancer	Epilepsy
Heart problems / angina	Respiratory problems	Uncontrolled high blood pressure	Other		

PARTICIPANT DETAILS

(S: At start/baseline assessment, L: At latest data collection point)

	Overall<br>(if one cohort)	Between groups (if comparing)		
Number at the start *(S) and latest **(L) data collection: no (%)	S:<br><br>L:			
Male/Female: no (%)	S:<br><br>L:			
Age in years:<br>(±SD, range)	S:<br><br>L:			
Duration of shoulder symptoms (±SD, range)<br>Indicate Yrs, mo, wks	S:<br><br>L:			

PT: Physiotherapy

*Where possible *start number* (S) refers to the number of participants for whom baseline data was collected and who also started physiotherapy (rather than number recruited to the study). If this information is not explicitly available please calculate it (and indicate as such) or provide whatever figure is available closest to this (and indicate as such).

**Where possible latest number (L) refers to the number or proportion of participants for whom baseline data was collected and also started treatment. Criteria and strategies if not available as for *start.

PHYSIOTHERAPY TREATMENT

What percentage of patients actually received the Physiotherapy intervention *at the start of the study*?

Not stated	% participants offered PT treatment	% participants received at least one session of the PT treatment	Other

How many times were patients seen by a Physiotherapist?

Not stated	Once	Twice	3-6 times	7-12 times	>12 times
Until symptoms resolved	Physiotherapists discretion	Other (please state)			

How long was each physiotherapy appointment?

Not stated	As required	≤ 15 minutes	16-30 minutes	31-60 minutes	> 1 hour

How long did the course of Physiotherapy last?

Not stated	As required	≤ 2 weeks	3-6 weeks	7-12 weeks	> 12 weeks

What treatments were prescribed or delivered by the physiotherapist(s)? (if more than one group indicate)

Not stated	Advice	Home exercises	Supervised exercises	Class	Hydrotherapy
Acupuncture	Injection	Shoulder Taping	Electrotherapy, heat and ice	PNF	Hands on scapula setting/ muscle recruitment/ or symptom modification
Manual therapy to spine	Manual therapy to shoulder	Stretches - Physio applied	Stretches Patient applied	Deep trans. frictions	
Other (please state)					

(If additional details available highlight page no and paragraph)..

COMPLIANCE WITH PHYSIOTHERAPY TREATMENT

How many of the original participants completed their course of Physiotherapy (relate to page 4)?

Not stated	N (%) completing with full attendance	N (%) completing with some attendance

Reasons for not completing course of physiotherapy

Not stated	Unknown	Details (state with no & %)

Did participants complete an exercise adherence diary (or similar)?

Not stated	No	Yes, but no further details	Yes (provide details)

Additional comments with respect to adherence to Physiotherapy treatment
(If details available highlight page no and paragraph)...

NON PHYSIOTHERAPY TREATMENT

Was Physiotherapy delivered as a part of a package of care involving other Health Care Professionals/Medics etc?

Not stated	No	Yes, Physiotherapy delivered with the following:					
		Medication from GP/Medic/Nurse Practitioner/Over the Counter/or Pharmacist	Pain management programme	Injection or hydrodilatation not delivered by a Physio	Sports Rehabilitation Team	Work related Rehabilitation Team	Other (Specify)

Were participants included if they were receiving treatment for their shoulder from other health professions?
INC if they were included, EXC if they were excluded, leave blank if not discussed. If *none* discussed, tick first option.

	Not stated	"Any other treatment"	Analgesic s/NSAIDS	Physio	Surgery	MUA	Steroid Inject'n	Hydro-dil'n	Pain clinic	Other (Specify)
Prior to the study; timescale if applicable										
At recruitment (and during Physio)										
Did some additional treatments occur anyway during Physio										
Between Physio and follow up										

Where any treatments prescribed by but not delivered by a physiotherapist?

Not stated	Unclear	No	Yes (details)

OUTCOME MEASURES

	√	Measurement tool	Comments
Pain - Score			
Pain - Other			
Global Rating Scale/Recovery/Presence or not of symptoms<br>(may relate to severity)			
Self rated functional/disability			
Adverse outcomes			
Constant score			
Other clinician rated shoulder score			
Mixed patient and clinical rating score			
Quality of life			
Time off/return to work			
Active range of shoulder movement			
Passive physiological range of shoulder movt.			
Shoulder strength			

Legend: Y=Yes, N=No, NS=Not stated,

At what timescales were outcome measures collected after the first physiotherapy appointment?

Not stated	< 4 weeks	4-8 wks	9-16 wks	5-8 mo	9-12 mo	16-20 mo	2 year +	Other
At discharge		Time to recovery (detail)						

What was the setting for collecting outcome data?

Not stated	Postal questionnaire	Telephone appointment	Routine follow up – Physio	Routine follow up – Medical	Specific appt for study	Other

Was the person collecting outcome data blinded to baseline/prognostic data or grouping?

Not stated	Yes	No	Not applicable (Patient rated)

Was all outcome data related to pre –defined prognostic factors?

Potential prognostic factors not defined a priori	Yes, all results reported	Yes, but only significant results reported	No, just related to selected outcome measure(s)	Unclear	Other

NUMBERS COMPLETING AND LOSS TO FOLLOW UP

How many of the original participants in the cohort/ RCT group were followed up?

Not stated	At first follow up: N (%) completing	At final follow up: N (%) completing

Were attempts made to collect outcome data on participants who were lost to follow up?

Not applicable	Not stated	No	Yes (but no details of how)	Yes, with details

Reasons for loss to follow up (circle correct response)

Not applicable	Not stated	Unknown	Deceased	Other (state with no & % for different timescales)

Are Participants for whom there is no outcome data compared with responders for prognostic features relevant to the study?

Not applicable	Not stated	No	Yes (no details)	Yes, with details

(If available highlight page no and paragraph)..

Are there differences between:
a) Prognostic factors under investigation and/or
b) Outcomes in participants *who completed physiotherapy and those who did not?*

	Not applicable	Not stated	No	Yes (but no details of how)	Yes, with details
Prognostic Factors					
Outcomes					

(If available highlight page no and paragraph)..

QUESTIONS FOR SPECIFIC STUDY DESIGNS

For studies comparing groups receiving the "same" physiotherapy treatment with potentially *different prognostic factors at baseline*:

a) Was there potential for selection bias between groups in terms of prognostic factors?

Not applicable to this study	Not applicable, this was part of an RCT	No	Yes, Clearly	Yes, but steps taken to minimise
Not stated	Other Comments			

b) Were there other potential differences between participants?

Not applicable						
Not stated	Yes, groups clearly not matched based on other factors	No, groups clearly matched based on other factors	"No difference between groups" stated in text	"Differences not statistically significant" stated in text	Some differences (confounders) are adjusted for in the analysis	All differences (confounders) are adjusted for in the analysis

Additional comments...

c) Was the physiotherapy delivered comparable between those with different prognostic factors?

Not applicable						
Not stated	Yes, one cohort received detailed standardised treatment	Yes, ≥2 groups received detailed standardised treatment	Treatment tailored to individual participant by physiotherapist	Details of potential differences not presented	Clear potential for differences from the description in the text	Other (please state)

Comments...
