# Supplementary material for: Predicting response to physiotherapy treatment for musculoskeletal shoulder pain: a systematic review
Source: BMC Musculoskelet Disord. 2013 Jul 8;14:203. doi: 10.1186/1471-2474-14-203 (PMC3717132; doi:10.1186/1471-2474-14-203)
Supplement: Additional file 3 — Criteria for the assessment of external validity, risk of bias, and presentation of results specific to the objectives of this review. [file 1471-2474-14-203-S3.pdf]

Additional File 3: Criteria for the assessment of external validity, risk of bias, and presentation of results specific to the objectives of this review.

Criteria Code	Description	Rating Options	Definition	Source
A	Participants invited onto the study representative of the population from which they were recruited	Yes, No, Unable to determine	The study must identify the source population for patients and describe how the patients were selected. All appropriate patients from the source population that fulfil the eligibility criteria should have an equal chance of being invited onto the study. Patients would be representative if they comprised the whole population, consecutive patients, or a random sample. Random sampling should be rigorous in its application.	Main source: Downs and Black 1998 qu. 11 (with additional comments from review team). Also informed by: Hayden 2006 domain 1, Wells 2011 "Selection" qu. 1 and 2
B	Reproducible selection criteria	Yes, No, In part	In cohort studies and trials, inclusion and exclusion criteria should be given such that findings can clearly and unambiguously be applied to specific patients in clinical practice. For this study, exclusion of patients with referral of pain from the cervical spine (in addition to radiculopathy) is essential. If all other criteria but this is covered, the answer should be in part.	Informed by: Downs and Black 1998 qu. 3; Hayden 2006 domain 2; Verhagen 1998 qu 1; Kuijpers et al 2004 criteria B;
C	Participants recruited onto the study representative of the population from which they were recruited	Yes, No, In part, Unable to determine	The proportion of those who were asked and agreed should be stated. Validation that the sample was representative would include demonstrating that the distribution of the main confounding variables was the same in the study sample and the source population. Where some differences do exist but are made explicit this should be answered IP. Where presentation concentrates on eligibility and does not record (potential) differences between consenters and non consenters this should be recorded as No.	Main source: Downs and Black 1998 qu. 12 (with additional comments from review team). Also informed by: Hayden 2006 domain 3, Wells 2011 "Selection" qu 1 and 2
D	Standardised, accurate assessment of baseline data which are being analysed as potential prognostic factors	Yes, No, Unable to determine	This should be answered Yes when the source of baseline data and how it was accessed is described such that a judgement can be made that the process was reproducible and data collected accurate. This should be answered No when the process described indicates the potential for miss-categorisation of potential prognostic factors. This should be answered Yes when baseline data being measured are less open to miss-categorisation - for example age, sex, handedness	Informed by: Hayden 2006 domain 7; Kuijpers et al 2004 criteria L and M
E	Standardised accurate outcome measure	Yes, No, Unable to determine	This should be answered Yes where outcome measures are clearly described and reproducible, without ambiguity of measures or categories. This includes when references are made to other studies which have demonstrated the accuracy of the outcome measure.	Informed by: Downs and Black 1998 qu. 20; Hayden 2006 domain 9; Kuijpers et al 2004 criteria K

F	Outcome assessor blinded to baseline data	Yes, No, Not applicable, Unable to determine	Was the outcome assessor blinded to baseline measures of potential predictive factors? This should not be confused with blinded to interventions in RCTs. Not applicable when the outcome is a patient rated questionnaire.	Informed by: Verhangen qu 7; Wells 2011 "Outcome 1"
G	All prognostic factors defined a-priori	Yes, No	Are all potential predictive factors (against which outcome measures will be analysed) clearly described prior to the results section? This is usually in the methods section.	Informed by: Downs and Black 1998 qu. 16; Hayden 2006 domain 6
H	All prognostic factors reported	Yes, No, Unable to determine	Are all predictive factors described a-priori, reported in the results section? If they are, irrespective of the standard of reporting, this should be answered as yes. If potential prognostic factors are not described a priori this should be answered unable to determine	Informed by: Hayden 2006 domain 6,
J	All outcome measures defined a-priori	Yes, No	Are all the outcomes to be measured (against which baseline potential predictive factors will be analysed) clearly described prior to the results section?	Informed by: Downs and Black 1998 qu. 2 and 16; Hayden 2006 domain 8
K	All outcome measures reported	Yes, No, Unable to determine	Are all of the outcomes which are described a-priori, reported in the results section with reference to the relevant potential prognostic variables? If all outcomes are reported this should be answered Yes irrespective of the standard of reporting. If outcome measures are not described a priori this should be answered as unable to determine	Source: Review Team
L	≥ One session of PT completed	Yes, No, Unable to determine	Evidence that ≥75% of participants received at least one session of physiotherapy?	Source: Review Team
M	Full PT attendance	Yes, No	Was the proportion of participants completing the full course of physiotherapy stated?	Source: Review Team
N	Compliance with home exercises	Yes, No, Unable to determine	Was the proportion of participants completing their prescribed home exercises reported? This should only be answered Yes if actual results are presented. Studies which state that exercise diaries were provided and/or compliance monitored but do not provide results, should be answered unable to determined	Source: Review Team
O	Absence of contamination by receiving treatment other than PT ± package defined at onset	Yes, No, Not stated	Where participants received additional treatments to Physiotherapy, other than those defined at onset, this should be answered No. When participants cross over from a non-physiotherapy treatment group to the physiotherapy treatment group this should be stated as No. Where cross over did occur but was minimal and explicitly accounted for in the results this should be stated as Yes.	Source: Review Team

P	Baselines differences between participants completing and not completing PT reported	Yes, No, Not applicable	This should be answered "not applicable" where all participants completed the full course of physiotherapy or those not completing were so small that findings would be unaffected by their inclusion. This should be answered no when a study does not report the number of patients not completing the full course of physiotherapy	Main Source: Downs and Black 1998 qu. 2 (with additional comments from review team).
Q	Loss to follow up for final outcome measure reported	Yes, No	If this is unclear, or ambiguous this should be reported as No. State loss to follow up in percentage.	Informed by: Downs and Black 1998 qu. 26; Hayden 2006 domain 4; Wells 2011 "outcome qu 3"
R	Baseline differences between participants with OC data and those lost to FU reported	Yes, No, Not Applicable	This should be answered "not applicable" where there were no losses to follow up or losses to follow up were so small that findings would be unaffected by their inclusion. This should be answered no when a study does not report the number of patients lost to follow up	Informed by: Hayden 2006 domain 5, Kuijpers et al 2004 criteria I, Wells 2011 "outcome qu 3"
S	Estimates of the random variability of data for the main outcome(s) presented	Yes, No, In part	In non-normally distributed data the inter-quartile range of results should be reported. In normally distributed data the standard error, standard deviation or confidence intervals should be reported. Where studies have presented change data within groups/groupings rather than differences between groups/groupings with different prognostic factors this should be stated as In part.	Main source: Downs and Black 1998 qu. 7 (with additional comments from review team). Also informed by Verhagen 1998 qu 10
T	Actual probability values reported for main outcome(s) irrespective of their value, except where ≤ 0.001	Yes, In part, No	Where studies have presented change data within groups/groupings rather than differences between groups/groupings with different prognostic factors this should be stated as In part. i.e. 0.035 rather than <0.05, to allow visualisation of trends when studies are underpowered.	Main source: Downs and Black 1998 qu. 10 (with additional comments from review team).
U	Adverse events reported or acknowledged	Yes, No	This should be answered yes if the study demonstrates that there was a comprehensive attempt to measure adverse events.	Source: Downs and Black 1998 qu. 8
V	No. of participants worsening with PT reported	Yes, No	This should be answered yes if the study states the number or proportion of participants whose outcome got worse during treatment.	Source: Review Team
W	Adequate adjustment for other confounding prognostic factors	Yes, No,	If this unclear, or ambiguous this should be reported as No. Where only uni-variate analysis has taken place a clinical judgement may be required to assess whether other factors should have been considered as potential confounders.	Informed by: Downs and Black 1998 qu. 25; Hayden 2006 domain 11

X	Measures of association (or difference between groups) stated		This should be answered Yes if the statistical measure of association or differences between groups reported (e.g. x^2, R^2, Beta or Odds ratio)	Informed by: Hayden 2006 domain 12
Y	Clear presentation and consistency of reporting	Yes, No	If this is unclear, or ambiguous this should be reported as No.	Informed by: Hayden 2006 domain 12, 13, 14; Downs and Black 1998 qu. 6
