# Supplementary material for: Predicting response to physiotherapy treatment for musculoskeletal shoulder pain: a systematic review
Source: BMC Musculoskelet Disord. 2013 Jul 8;14:203. doi: 10.1186/1471-2474-14-203 (PMC3717132; doi:10.1186/1471-2474-14-203)
Supplement: Additional file 4 — Engebretson et al’s [23] multiple regression model (backward) predicting SPADI at one year follow up and synopsis of uni-variate analysis (n = 104). [file 1471-2474-14-203-S4.pdf]

Additional file 4: Engebretson et al's [23] multiple regression model (backward) predicting SPADI at one year follow up and synopsis of uni-variate analysis (n=104)

Outcome	Variable	ß	95% CI	p-value
SPADI at one year follow up	Less than 12 years in school (no, yes)	-14.3	-23.5, -5.2	0.003
	Previous shoulder pain (no, yes)	11.0	1.4, 20.6	0.026
	Baseline SPADI (High score=high disability)	0.37	0.15, 0.59	0.001
Adjusted for age, gender and treatment group. Total R^2 29.9%				
Factors significant on uni-variate analysis p<0.1:				
Work status, Duration of pain 6-12 months, distress (HSCL 25), health status (EQ-VAS), pain at rest, self-efficacy for pain, previous shoulder pain, active range of shoulder flexion and hand behind back				
Factors insignificant on uni-variate analysis p≥0.1:				
Gender, Age, duration of pain 3-6 months and >12 months, previous physiotherapy, medication, neck pain.				
