# Supplementary material for: Predicting response to physiotherapy treatment for musculoskeletal shoulder pain: a systematic review
Source: BMC Musculoskelet Disord. 2013 Jul 8;14:203. doi: 10.1186/1471-2474-14-203 (PMC3717132; doi:10.1186/1471-2474-14-203)
Supplement: Additional file 5 — Deutscher et al’s [31] multiple regression analyses predicting functional status, (CAT) [43] at discharge. [file 1471-2474-14-203-S5.pdf]

Additional file 5: Deutscher et al's [31] multiple regression analyses predicting functional status, (CAT) [40] at discharge.

Outcome	Variable	ß	T	P
	Constant	40.6	31.9	<0.001
Deutscher (n=5252) Functional status (CAT) at discharge.	Age	-0.1	-8.0	<0.001
	Female	-2.2	-6.1	<0.001
	Language=Hebrew	0.9	2.5	0.013
	Payer=Maccabi	4.0	7.2	<0.001
	Duration of symptoms <21 days	2.2	4.2	<0.001
	Duration of symptoms > 90 days	-2.2	-5.9	<0.001
	Intake functional status	0.5	33.4	<0.001
	Physical activity 1-2x per week	1.1	2.5	0.012
	Cardiovascular registry	-1.1	-2.0	0.042
	Tobacco use disorder	-1.4	-2.1	0.035
	Antidepressant medication	-1.8	-3.5	<0.001

Factors not significant on multiple regression analysis:

Daily activity=office, Language =Russian, Payer=work, Medication use at intake, No of related surgeries=1 or more, Physical activity=none, No of comorbidities, No of medical registries, Asthma, Migraine, Osteoporosis
