# Supplementary material for: Predicting response to physiotherapy treatment for musculoskeletal shoulder pain: a systematic review
Source: BMC Musculoskelet Disord. 2013 Jul 8;14:203. doi: 10.1186/1471-2474-14-203 (PMC3717132; doi:10.1186/1471-2474-14-203)
Supplement: Additional file 6 — Kim et al’s [17] functional outcomes for groups with and without a painful jerk test [41]. [file 1471-2474-14-203-S6.pdf]

Additional file 6: Kim et al’s [17] functional outcomes for groups with and without a painful jerk test [41]

Outcome	Group with a Painless Jerk Test n=48			Group with a Painful Jerk Test n=33		
	Baseline Mean±SD (95% CI)	Follow up Mean±SD (95% CI)	P value	Baseline Mean±SD (95% CI)	Follow up Mean±SD (95% CI)	P value
UCLA	24±4 (23-25)	33±3 (32-34)	<0.001	23±3 (22-24)	27±5 (25-28)	<0.001
ASES	68±13 (65-72)	92±8 (90-95)	<0.001	65±12(61-69)	72±16 (67-78)	0.038
ROWE	50±11 (47-53)	93±10(90-96)	<0.001	52±12(48-56)	69±15 (64-75)	<0.001

Legend:

UCLA Rating system of the University of California at Los Angeles [43]

ASES The American Shoulder and Elbow Surgeons Shoulder Index [44]

Rowe The rating system of Rowe et al [42]
