# Supplementary material for: Predicting response to physiotherapy treatment for musculoskeletal shoulder pain: a systematic review
Source: BMC Musculoskelet Disord. 2013 Jul 8;14:203. doi: 10.1186/1471-2474-14-203 (PMC3717132; doi:10.1186/1471-2474-14-203)
Supplement: Additional file 7 — Accuracy statistics for the factors retained within Yang et al’s [19] clinical predication rule (for a positive outcome). [file 1471-2474-14-203-S7.pdf]

Additional file 7: Accuracy statistics for the factors retained within Yang et al's [19] clinical predication rule (for a positive outcome)

	Improvement n=14 Mean±SD	Non improvers n=20 Mean±SD	P value
Humeral elevation (°)	102.5±19.9	89.3±18.0	0.057*
External rotation (hand to neck) (°)	52.3±26.6	39.9±16.2	0.097*
Internal rotation (hand to back) (°)	15.6±11.1	11.0±5.5	0.121*

*Independent sample t test

	Sensitivity Mean (95% CI)	Specificity Mean (95% CI)	+ve LR
Humeral elevation > 97.0°	71.4 (41.9-91.4)	70.0 (45.7-88.0)	2.08
External rotation (Hand to neck) > 38.9°	71.4 (41.9-91.4)	60.0 (36.1-80.8)	2.21

N.B. Kinematic variables investigated by FASTRAK 3-D electromagnetic capturing systems (Polhemius Inc, Cochester, VT, USA) rather than clinical examination are not reported in this review.
