# Supplementary material for: Predicting response to physiotherapy treatment for musculoskeletal shoulder pain: a systematic review
Source: BMC Musculoskelet Disord. 2013 Jul 8;14:203. doi: 10.1186/1471-2474-14-203 (PMC3717132; doi:10.1186/1471-2474-14-203)
Supplement: Additional file 8 — Kennedy’s [32] multiple regression models (n = 289) for DASH scores at discharge or 12 weeks and synopsis of uni-variate analysis. [file 1471-2474-14-203-S8.pdf]

**Additional file 8: Kennedy's [32] multiple regression models (n=289) for DASH scores at discharge or 12 weeks and synopsis of uni-variate analysis**

| **Predictors of higher log (1+DASH score), or greater disability at discharge or 12 weeks.** | | | | | | |
|---|---|---|---|---|---|---|
| | ß | SE | 95%CI | P | Standardised ß | Partial $R^2$ |
| Higher DASH (more disability) | 0.02 | 0.002 | 0.01, 0.03 | <0.0001 | 0.40 | 0.20 |
| Covered by workers compensation claim | 0.55 | 0.19 | 0.18, 0.92 | 0.0036 | 0.16 | 0.08 |
| Therapist predicts more restriction of activities at DC | 0.42 | 0.10 | 0.21, 0.63 | <0.001 | 0.21 | 0.04 |
| Older age (decades) | 0.13 | 0.03 | 0.06, 0.19 | 0.0004 | 0.19 | 0.03 |
| Female | -0.24 | 0.10 | -0.45, -0.04 | 0.0219 | -0.12 | 0.01 |
| *Additional factors significant on uni-variate analysis (p≤0.1)* | Age, No of co-morbidities and No limiting activity, Recurrent problem, Pain intensity, Previous surgery, Current work status, Patient global rating of problem, Physical and Mental SF-36 score, Over the counter and prescription medication use, Restriction of normal range of movement (normal or some), Muscle strength (Normal or some decrease), Patient prediction of recovery and estimate of time of return to work. Therapist prediction of time to return to activity | | | | | |
| *Additional factors insignificant on uni-variate analysis (p>0.1)* | Sudden onset, Duration of shoulder pain, Muscle wasting (Normal or some), | | | | | |

| **Predictors of greater reduction (improvement) in DASH score at discharge or 12 weeks.** | | | | | | |
|---|---|---|---|---|---|---|
| | ß | SE | 95%CI | P | Standardised ß | Partial $R^2$ |
| Higher pain intensity | -1.56 | 0.5 | -2.56, -0.57 | 0.02 | -0.2 | 0.08 |
| Surgery in the last 6 months | -15.73 | 3.46 | -22.55, -8.9 | <0.0001 | -0.26 | 0.07 |
| Shorter duration of shoulder pain | 4.04 | 1.24 | 1.60, 6.47 | 0.001 | 0.19 | 0.04 |
| Younger age (decades) | 1.58 | 0.7 | 0.21, 2.96 | 0.0243 | 0.13 | 0.02 |
| Lower physical component score on SF-36 (poorer health) | 0.25 | 0.13 | 0.01, 0.05 | 0.0448 | 0.13 | 0.01 |
| *Additional factors significant on uni-variate analysis (p≤0.1)* | Recurrent problem, Current work status, Patient global rating of problem, Mental SF-36 score, Over the counter and prescription medication use, Restriction of normal range of movement (normal or some), Muscle strength (Normal or some decrease), Restriction of normal range of movement (normal or some), Patient prediction for recovery | | | | | |
| *Additional factors insignificant on uni-variate analysis (p>0.1)* | Gender, No of co-morbidities and No limiting activity, Sudden onset, workers compensation, Muscle wasting (None, some), Therapists prediction of activity and therapist and patients estimate of time of return to activity | | | | | |
