# Supplementary material for: Predicting response to physiotherapy treatment for musculoskeletal shoulder pain: a systematic review
Source: BMC Musculoskelet Disord. 2013 Jul 8;14:203. doi: 10.1186/1471-2474-14-203 (PMC3717132; doi:10.1186/1471-2474-14-203)
Supplement: Additional file 9 — Accuracy statistics for the factors retained (and synopsis of those excluded) within Mintken et al’s [21] and Hung et al’s [20] clinical predication rules (for a positive outcome). [file 1471-2474-14-203-S9.pdf]

Additional file 9: Accuracy statistics for the factors retained (and synopsis of those excluded) within Mintken et al's [21] and Hung et al's [20] clinical predication rules (for a positive outcome).

Outcome	Variable	Sensitivity (95% CI)	Specificity (95% CI)	+ve LR (95% CI)	Posttest Prob'y %
Mintken [21] (n=80) ≥4 GROC after 1-3 treatments	Shoulder pain less than 90 days	0.47 (0.33, 0.62)	0.84 (0.66, 0.94)	2.9 (1.2, 6.6)	81.9
	No medication for shoulder pain	0.38 (0.24, 0.53)	0.83 (0.65, 0.94)	2.3 (0.93, 5.4)	78.3
	Painfree active sh. flexion <127°	0.59 (0.44, 0.73)	0.74 (0.55, 0.87)	2.3 (1.2, 4.4)	78.3
	Negative neer	0.50 (0.35, 0.65)	0.73 (0.54, 0.87)	1.9 (0.97, 3.6)	74.8
	IR <53° at 90° abduction	0.78 (0.63, 0.88)	0.53 (0.35, 0.71)	1.7 (1.1, 2.5)	72.7
Pretest probability of success 61%. Nagelkerte R^2=0.56, Hosmer-Lemeshow p=0.90					
Factors which were significant different between groups p≤0.1			BMI, Symptoms distal to the shoulder,		
Factors which were not significantly different between groups p>0.1			Age, gender, Pain intensity, SPADI score, fear avoidance beliefs questionnaire, Tampa scale of kinesiophobia, Prior history of shoulder pain, Traumatic injury		

Outcome	Variable	Sensitivity (95% CI)	Specificity (95% CI)	+ve LR (95% CI)	Posttest Prob'y (%)
Hung [20] (n=33) ≥4 GROC at 6 weeks	FLEX-SF <41	100 (85, 100)	50 (18.9, 81.1)	2.00	UTD
	Serratus anterior <27.4% weight	91.30 (71.9, 98.7)	50 (18.9, 81.1)	1.83	UTD
Model X^2=23.71, df=3, p<0.0001, Nagelkerke R^2 = 0.725					
Factors which were significant different between groups p≤0.1		External rotation (% weight),			
Factors which were not significantly different between groups p>0.1		Age, Height, Weight, Symptom duration, Flexion (°), Abduction (°), Internal rotation (°), External rotation (°), Internal rotator (% weight), Abductor (% weight), Lower trapezius (% weight),			

UTD: Unable to determine
