# Supplementary material for: Predicting response to physiotherapy treatment for musculoskeletal shoulder pain: a systematic review
Source: BMC Musculoskelet Disord. 2013 Jul 8;14:203. doi: 10.1186/1471-2474-14-203 (PMC3717132; doi:10.1186/1471-2474-14-203)
Supplement: Additional file 10 — Predictive Factors for Pain: Kim et al’s [17] and Ryall [16] [Personal communications: Unpublished data. Palmer K and Ntani G, University of Southampton, 2012]. Odds ratios and 95% confidence intervals for statistically significant factors (p≤0.5) and synopsis of results not reach significance (p>0.5). [file 1471-2474-14-203-S10.pdf]

Additional file 10: Predictive Factors for Pain: Kim et al's [17] and Ryall [16] [Personal communications: Palmer K and Ntani G, Unpublished data. University of Southampton, 2012]. Odds ratios and 95% confidence intervals for statistically significant factors (p≤0.5) and synopsis of results not reach significance (p>0.5).

Kim et al: [17] Difference in pain scores between participants with & without a painful jerk test

	Group with Painless Jerk Test n=48			Group with a Painful Jerk Test n=33		
Outcome	Baseline Mean±SD (95% CI)	Follow up Mean±SD (95% CI)	P value	Baseline Mean±SD (95% CI)	Follow up Mean±SD (95% CI)	P value
Mean Pain Score (VAS)	3.6±3.1 (3.2-3.9)	0.2±0.5 (0.1-0.4)	<0.001	3.5±1.4 (3.0-4.0)	2.3±1.8 (1.7-3.0)	<0.008

Ryall [16], Palmer and Ntani [Personal Communications]: Unpublished data. Odds ratios and 95% confidence intervals for statistically significant factors (p≤0.5) and synopsis of results not reaching statistical significance (p>0.5).

	Pain present in the last 4 weeks		Pain present ≥14 days in last 4 weeks		Pain present every day for last 7 days	
Risk factor n=132	N (%)	OR (95% CI)	N (%)	OR (95% CI)	N (%)	OR (95% CI)
Non smoker n=103	52 (51)	Baseline	21 (20)	Baseline	22 (21)	Baseline
Current smoker n=27	17 (63)	1.5 (0.6, 4.1)	12 (44)	2.4 (0.8, 7.6)	10 (37)	2.7 (1.0, 7.2)
Chronic widespread pain n=20	14 (70)	2.4 (0.8, 7.4)	6 (30)	2.1 (0.6, 7.6)	9 (45)	2.9 (1.0, 8.4)
**Odds ratio ≥ 2*	Bilateral versus unilateral shoulder pain, More than 14 days of pain in the last month at baseline, Whitely hypochondriasis score 5+, hospital anxiety and depression (depression) score 8+, somatising tendency score 3, chronic widespread pain, chronic abdominal pain, beliefs relating physical activity to arm pain					
**Odds ratio < 2*	Sex, age, disabling pain in the last month at baseline, disabling pain in the last month at baseline, hospital anxiety and depression (anxiety) score 8+, somatising tendency score ≤ 2, chronic headache, chronic fatigue syndrome, occupation (manual, unemployed versus non manual), belief that problem is likely to be causing difficulties 3 months later.					

*95 Confidence intervals passed through 1 to varying degrees. Numbers in some categories were small.
