# Supplementary material for: Predicting response to physiotherapy treatment for musculoskeletal shoulder pain: a systematic review
Source: BMC Musculoskelet Disord. 2013 Jul 8;14:203. doi: 10.1186/1471-2474-14-203 (PMC3717132; doi:10.1186/1471-2474-14-203)
Supplement: Additional file 11 — Predictors for time off work, Engebretson’s [23] logistic regression model (forwards) and Mintken’s [21,69] predictive statistics. [file 1471-2474-14-203-S11.pdf]

Additional file 11: Predictors for time off work, Engebretson's [23] logistic regression model (forwards) and Mintken's [21,69] predictive statistics

			ß	OR	95% CI	p-value
Engebretsen [23] Work status at one year* N=90, 23 (25%) off work.	Less than 12 years in school (no, yes)		1.5	4.3	1.3, 14.9	0.02
	Self-reported health status (EQ-VAS). Higher score=better health		0.06	1.06	1.0, 1.1	0.001

*Adjusted for age, gender and treatment group.

Factors significant on uni-variate analysis p≤0.1:
Work status, self-efficacy for pain, baseline SPADI, previous shoulder pain, previous physiotherapy, active range of shoulder flexion and hand behind back
Factors insignificant on uni-variate analysis p>0.1:
Gender, Age, duration of pain 6-12 months, 3-6 months and >12 months, medication, distress (HSCL 25), pain at rest, neck pain.

Mintken [21,69]	Missing work during 48 hrs		AUC	95% CI
N=80, 9 (11%) missed work	Higher score=higher fear avoidance beliefs	FABQ (WB)	0.75	0.54, 0.96
		FABQ (PA)	0.57	0.34, 0.81
