# Supplementary material for: Predicting response to physiotherapy treatment for musculoskeletal shoulder pain: a systematic review
Source: BMC Musculoskelet Disord. 2013 Jul 8;14:203. doi: 10.1186/1471-2474-14-203 (PMC3717132; doi:10.1186/1471-2474-14-203)
Supplement: Additional file 12 — Tanaka’s [18] comparison of outcome (improvement in abduction) for each category. [file 1471-2474-14-203-S12.pdf]

Additional file 12: Tanaka's [18] comparison of outcome (improvement in abduction °) for each category.

Variable	Variable categories and number of participants	Improvement in abduction (°) Mean±SD (95%CI)	Comparator	P Value
Age	50 and below (n=11)	70±15 (60-80)	Age 71 and above	0.001
	50s (n=27)	54±22 (45-63)		0.028
	60s (n=51)	54±27 (47-62)		0.010
	71 and above (n=21)	34±22 (24-44)		
Handedness	Dominant (n=60)	59±21	Dominant and non-dominant	0.002
	Non dominant (n=50)	43±28		
Gender	Male (n=52)	56±24	Male and female	NS
	Female (n=58)	47±27		
Duration of the condition	< 1 month (n=37)	48±27 (39-56)		
	< 3 months (n=39)	57±23 (49-64)	> 7 months	0.050
	< 6 months (n=21)	60±20 (51-70)	> 7 months	0.037
	> 7 months (n=13)	36±31 (17-54)		

NS Not significant
