# Supplementary material for: Predicting response to physiotherapy treatment for musculoskeletal shoulder pain: a systematic review
Source: BMC Musculoskelet Disord. 2013 Jul 8;14:203. doi: 10.1186/1471-2474-14-203 (PMC3717132; doi:10.1186/1471-2474-14-203)
Supplement: Additional file 13 — Studies [17,21,23,32,69] (n = 4) reporting numbers who get worse during physiotherapy or at follow up. [file 1471-2474-14-203-S13.pdf]

Additional file 13: Studies [17,21,23,32,69] (n=4) reporting numbers who get worse during physiotherapy or at follow up.

Engebretson 2010 [23]	Treatment	Supervised exercise group (n=52)	Extracorporeal shock treatment (n=52)
	No. (%) whose shoulder pain increased	1 (2%) Suspected adhesive capsulitis.	2 (4%) One crossed over to sup'd ex.
Mintken 2010 [21,69]	Treatment	Cervico-thoracic manipulation (n=80)	
	No. (%) whose Shoulder pain increased	3 (4%)	
Kim 2004 [17]	Treatment	Supervised and home exercises	
	Prognostic group	Painless Jerk Group (n=48, 54 shoulders)	Painful Jerk Group (n=33, 35 shoulders)
	No (5) with the same or worse outcome	4 (7%)	30 (84%)
Kennedy 2006 [32]	Treatment Group	Physiotherapy (no further details) n=289	
	No (%) who had increased DASH scores*	≤ 20 (7%)	

*Estimated by reviewers from charts published by original authors
